# 

**Published:** 1904-07-15

**Authors:** 


### ANNOUNCEMENTS.

DR. A. W. HARLAN has removed from Chicago to New York City, for the practice exclusively of dental therapeutics. He will specialize in the treatment of pyorrhœa alveolaris and other diseases of the mouth. It is a new undertaking, and Dr. Harlan will surely succeed if any one can. His friends in the West wish him all success, and we cordially commend him to any one requiring such services.

THE Interstate Dental Fraternity will meet at St. Louis, in annual session, Tuesday, August 30th, 1904, at 3 P. M. Banquet tickets can be had by addressing Dr. E. E. Haverstick, 346 N. Boyle Ave., St. Louis, Mo.

DR. ARTHUR D. BLACK has opened a dental journal clearing-house, by means of which he hopes to be able to sort out and distribute to people who are collecting dental libraries such dental journals as may come into his hands. Dentists who have old journals which they do not want will do a good thing to box them and ship to Dr. Black, care of Northwestern Dental College, Chicago.

THE Fourth International Dental Congress banquet will be held on September 1st, 1904, at eight P. M. in the Coliseum adjoining the Congress Hall. The price per plate is three dollars. It is requested that all who expect to attend send their names and money to Dr. A. H. Fuller, P. O. Lock Box 604, St. Louis, Mo., at once and not later than

August 20th. Arrangements to pay can be made with Dr. A. H. Fuller, at the time of registration, provided notice is given before August 20th.

G. A. Bowman,
A. H. Fuller,
Adam Flickinger,
*Banquet Committee.*

---

F. D. I.—Federation Dentaire Internationale.—The next (fourth annual) meeting will be held in the Coliseum Building, St. Louis, Mo., August 26th and 27th.

The first session will convene under the presidency of Dr. Charles Godon, at 11 A. M. There will be a meeting of the Executive Council on Thursday, the 25th, at the Hotel Jefferson, at 10 A. M.

The section on Education will meet at 3:00 P. M., Friday.

The section on Hygiene and Public Dental Service, will meet at 3:00 P. M., Friday.

The section on International Dental Press will meet at 4:30 P. M., Friday.

The officers of the section are, Education: President, Dr. T. W. Brophy; Vice-Presidents, Dr. E. C. Kirk, Dr. W. B. Paterson and Dr. O. Zsigmondy; Secretaries, Dr. M. Roy and Dr. R. B. Weiser.

Hygiene and Public Dental Service: President, Dr. W. D. Miller; Vice-Presidents, Dr. Cunningham, Dr. Forberg, Dr. Jenkins and Dr. Rose; Secretaries, Dr. R. Heide, Dr. Sauvez and Dr. R. B. Weiser.

Commission on International Dental Press: President, Dr. E. Forberg; Vice-President, Dr. A. W. Harlan; Secretary, Dr. E. Papot.

Executive Council: President, Dr. Charles Godon; Vice-Presidents, Dr. A. W. Harlan, Dr. W. D. Miller; Secretary, Dr. E. Sauvez; Treasurer, Dr. F. Aguilar.

Members: Dr. Geo. Cunningham, Dr. E. Forberg, Dr. R. B. Weiser, Dr. E. Grevers, Dr. F. Heide, Dr. Klingelhofer.

On behalf of the Federation:

A. W. HARLAN, *Vice-Pres.*

1122 Broadway, New York City.

### INTERNATIONAL CONGRESS.

THE Local Committee and Bureau of Information wish to dispel from the minds of the profession and all persons that labor under any misconception that the rates of St. Louis hotels are extortionate.

We have investigated the conditions and rates of the leading hotels of St. Louis, and notwithstanding the fact that this city is entertaining a World's Fair, the hotel rates are no higher than in other cities.

We will append a number of the leading hotels and rates of same and call to your attention that it is not required of you to put up at any of the hotels mentioned, as there are many hotels and boarding houses in the city where rooms can be secured for from 50 cents to $2.00 per day. The exact date should be stated in securing rooms.

We append a list of hotels and rates of same as follows:

SOUTHERN HOTEL. The American plan rate is $5.00 per day for room without bath and $6.00 a day for a room with bath. The rate is $10.00 per day if two persons occupy the room.

PLANTERS' HOTEL. Room without bath, occupied by one person, $3.00 to $4.00 a day. Same for two persons, $6.00 to $7.00 a day. Room with bath for one person, $4.00 to $5.00 a day, and for two persons $7.00 to $8.00 a day.

JEFFERSON HOTEL. Room without bath, for one person,

$4.00 a day; when occupied by two persons, $6.00 a day. Room with bath for one person, $5.00 a day and up; when occupied by two or more persons, $7.00 a day and up.

St. Nicholas Hotel. Room without bath, for one person, $2.50 to $3.50 a day, for two persons, $4.00 to $5.00 a day. Room with bath, for one person, $3.00 to $5.00 a day; for two persons, $5.00 to $7.00 a day.

Lindell Hotel. Room without bath, for one person, $2.00 a day; for two persons, $3.00 a day. Room with bath, for one person, $3.00 a day; for two persons, $4.00 a day.

Washington Hotel. Room without bath, for one, two or three persons, $5.00 to $7.00 a day; room with bath, for one, two, or three persons, $8.00 a day.

Laclede Hotel. Room without bath, for one person, $1.50 to $2.00 a day; for two persons, $3.00 a day; room with bath for one person, $3.00 a day; for two persons, $5.00 a day.

Terminal Hotel. Room without bath, for one person, $2.00 to $3.00 a day; for two persons, $4.00 to $5.00 a day. Room with bath, for one person, $5.00 a day; for two persons, $7.00 a day.

Mosier Hotel. $1.00 to $3.00 per day, European plan, with Silver Moon Restaurant attached, at very reasonable rates. Located at 9th and Pine Streets.

Hotel Rozier. Opposite Exposition Building, Olive and Thirteenth streets. Rooms without bath, $1.00 to $2.00 a day.

Mammoth Hotel Company. S. E. Corner Olive and Twelfth streets. Can accommodate 2,500 guests per day at rates from 50 cents to $1.50 per day.

The Inside Inn, with a capacity of 5,500 people, is within the Exposition grounds, erected under a contract with the Exposition Management, stipulating its rates.

This hotel offers 500 rooms at $1.00 per day, 500 at $1.50 a day, 500 at $2.00 a day, and the remainder which are larger, with baths, at higher rates. The Napoleon

Bonaparte, the Forest City, the Fraternal, the University, the Kenilworth, the American, the Epworth, the Grand View, the States, the Oakland, the Iowa, the Guaranty, the West Park, the Christian Endeavor, the Visitors, and others, with a capacity for from 500 to 5,000 guests are within easy walking distance of the World's Fair gates. In fact we have hotels, boarding houses, apartment houses, and rooming houses, all over the city, of respectable character and on the street-car lines.

An impression prevails that there may be lack of accommodation at reasonable prices. Not only will there be sufficient room for all who come, but the rates will be reasonable. We appeal to the profession of the country to give information respecting the accommodations in a spirit of fairness and justice to St. Louis based upon the above facts.

The membership fee to the Congress will be ten dollars. It will entitle its owner to the official badge and all rights and privileges of the Congress, also a copy of the printed transactions. It will be necessary to have this membership to get admission to the general meetings, sections, clinics, or any entertainments. Apply for your certificate at once to your State chairman. St. Louis is prepared to care for and welcome all comers and to show them the grandest Universal Exposition of the world's resources and products in the history of man.

The Fourth International Congress is now an assured success. Many foreign nations have signified their intention to take part in this Congress. The Islands of the Pacific Ocean will be well represented. Their assembling in the center of this great republic should stimulate every American dentist to action, and each individual of this great profession should feel under obligations to help to push the Congress to a successful issue. We have our professional record to maintain and act the part of host. As a consequence we should endeavor to sustain the reputation of American hospitality.

Here is the birthplace of the dental college, the most of the inventors, the mechanical geniuses, and the men who have brought about the wonderful advances in our great profession.

We trust the profession of America will take hold with its accustomed vigor, leave nothing undone that will make for the good of the profession, and carry it out with a most liberal spirit to a surprising conclusion.

A list of hotels, boarding houses, rooming houses and private homes with their rates appended will be furnished by the Committee to all asking for same. Any other information will be freely given by writing to Dr. D. O. M. LeCron, Chairman, Missouri Trust Bldg.

